# Notes on Puerperal Cases*Read before the Buffalo Medical Club, May 25, 1881.

**Published:** 1881-07

**Authors:** Thomas Lothrop


BUFFALO

MEDICAL AND SURGICAL JOURNAL.

VOL. XX.—JULY, 1881.—NO. 12.

### Original Communications.

### NOTES ON PUERPERAL CASES.*

BY THOMAS LOTHROP, M. D.

In a period of less than a year two fatal cases, occurring during the puerperal period, subsequent to an easy and natural labor, came within my professional observation. Inasmuch as these cases were, at the time, of great interest in a strictly medical point of view, it may not be presumptuous to make them the text upon which to present some practical considerations to the Club on certain diseases or conditions connected with the lying-in period. If any apology is desired for this digression from the course usually adopted in presenting papers for your consideration, the writer may state that deductions based upon experience obtained at the bedside, are not only instructive and valuable, but, if carefully and thoughtfully made, really constitute the only safe and reliable guide to those who, engaged in the responsible duties often devolving upon the obstetrician, may sooner or later encounter like difficulties, and witness like results.

*Read before the Buffalo Medical Club, May 25, 1881.

In a meeting such as this, assembled for mutual improvement in one of the most comprehensive studies in which the human intellect can test its capabilities, the bare statement of facts developed in the management of a disease, to which any one of us may, at a moment's notice, be summoned, may highten our sense of interest and awaken new ideas and clearer perceptions through even an imperfect presentation of the subject.

Permit me to preface my remarks by stating that in the present unsettled state of the pathology of many puerperal diseases, a wide latitude is allowed for their discussion. It is acknowledged by the best authorities that the diseases of the lying-in chamber, such as puerperal fever, septicæmia, pyæmia, and sapræmia, all of which are accompanied by fever and are attended by great mortality, merge into each other, and are often complicated in their progress with morbid phenomena belonging to one or more of the diseases above referred to. In the standard works, puerperal fever is most prominently brought to the attention of the reader, as it is the more frequently met with in practice than any other complication of the puerperal period. The diagnosis of these diseases is not always accurately made even by practitioners of large experience, on account of their ill-defined limitations.

Case 1. Mrs. W., aged 30 years, was taken with labor pains late in the evening of Saturday, Nov. 29, 1879. I was summoned soon after midnight; the patient was delivered of a fine, healthy female child at 6 A. M., Nov. 30th. The labor was natural, the placenta promptly expelled, and only the usual hemorrhage followed the delivery. This was her second confinement; the first, under one of our most experienced and judicious obstetricians, having been protracted and severe, and terminated only by the use of the forceps, the child surviving its birth only a few minutes. Very naturally, the present labor was looked forward to with the greatest apprehensions by the patient and her family. During the week following the delivery, the condition of the patient was as favorable as any I have ever attended: the lochia usually normal in quantity and quality; the milk profuse in quantity; the general symptoms favorable, so that my visits, being unnecessary, were irregularly made. On Sunday, Dec. 7, at 12 M., being the eighth day after her confinement, the patient had a severe chill, followed by a high fever. During the seven days following, the severer symptoms of puerperal fever were present, the morning temperature 101–2, the evening temperature 104–6; death occurred Dec. 14th, fourteen days after labor, and seven days after the primary chill In the management of the labor, and during the entire course of the disease, thorough antiseptic precautions and measures were adopted, the sanitary condition of the apartments was good, and the attendance of the nurse was constant and directed with good judgment.

CASE 2. Oct. 16, 1880, Mrs M. L D, aged 33 years, was delivered of a male child weighing, when dressed, 15½ lbs, after a natural and easy labor; the placenta was expelled by the uterine contractions, without traction upon the cord, in a brief period after the birth of the child. The pulse, an hour after delivery, was 120; ordered at once beef tea, milk, etc.

Oct. 16, 12 M. Pulse 120, temp 102; prescribed quinine sulp. gr. v every six hours, vaginal injections of carbolized water three times daily.

Oct. 17, 11 A. M. Pulse 112, temp. 102, lochia normal.

Oct 18, 11 A M. Pulse 108, temp 100; the secretion of milk ample to satisfy the new-born, and lochia light in color and muco-purulent in character.

Oct. 19 Pulse 100, temp 100; ordered quinine sulp. grs. v every eight hours.

Oct 20, 11 A. M. Pulse 100 temp. 100; at 12 M. the patient had a severe chill, but without pain in supra pubic region, even on severe pressure. 3 P. M. Pulse 140, temp 105; the lochia muco-purulent and without offensive odor; used intra-uterine injections of carbolic acid and warm water, 1—40, by means of a flexible catheter carried up to the fundus uteri, and connected with Davidson's syringe by means of a rubber tube; temperature fell to 103 within an hour after the injection, and to 102 at midnight Continued quiniæ sulp. gr. v every four hours, larger doses producing so uncomfortable a sensation in the head that the patient refused to take any additional quantity; ordered also during the pyrexia tr. aconite rad gtt j, liq. ammoniæ acetat ℥ ij every hour.

Oct. 21, 11 A. M. Temp. 105.5; used intra uterine injection of carbolized water with the same effect as in the preceding day; treatment continued

Oct. 22. Pulse 94, temp. 102; the highest temperature of the day, 104, attained at 4 P. M., falling at 10 P M. to 101. Used intra-uterine injections as before.

Oct. 23, 1 A M. Pulse 96, temp. 101; the maximum temperature of the day reached at 8 P. M., when the thermometer registered 103.5; treatment continued.

Oct. 24, 1 A. M. Temp. 102; 4 P. M, 99 5 and pulse 96. Temperature 100 at midnight.

Oct 25 Temperature ranged from 100 at 2 A. M. to 99.5 at 10 A. M, and 101.5 at 8 P. M.

Oct. 26, ten days after delivery, 6:20 A M. Temp 100; 3:30 P. M., 101.5; 10 P. M, 100.5.

Oct. 27, 1 A. M. Temp. 99, pulse 96; 4:30 A. M, patient had a severe chill; 10 A. M, temp. 105, pulse 140; used intra-uterine injections of carbolized water, 1—25; temperature fell to 102 at 2 P. M., and registered 105 at 5 P. M, falling to 100 with the pulse at 90 at 12 o'clock midnight, after the second intra-uterine injection.

Oct. 28. Temp. at 1 A. M., 100 5; 10 A. M., 99.5; 12 M patient had a chill, followed by rapid elevation of temperalure, at 2 P M. reaching 106 and after the intra-uterine injection falling to 102 at midnight Quinine tr. aconite with abundant nourishment were used.

Oct. 29, 2 A. M. Temp. 101 5. The highest temperature of the day being attained at 6 P. M, when the thermometer registered 104 5; treatment continued; added tr. ferri m. gtts. xv, and Potass chloratis grs x, every three hours.

Oct. 30, 1 A M Temperature 103.5; 5:20, 104; 9 P M, 105, and at midnight 104. Antipyretics discontinued, and the iron and Potass chloratis administered. Intra-uterine injections fail to diminish the fever heat.

Oct. 31, 6 A. M. 106.

Nov. 2, 10:30 P. M. Death.

Permit me to direct attention to the prominent features of these cases. Case 1 was an easy, natural labor, and for over seven days pursued an uninterrupted course towards a full and complete recovery, until my visits, seeming unnecessary, were irregularly made. The patient, the morning prior to the chill, which announced the invason of the fatal malady, was in the very best of spirits, with every prospect of a successful termination of the puerperal period. The causes, exciting and antecedent, leading to the onslaught of the disease, the writer has never been able to satisfactorily explain. The sanitary condition ot the habitation was examined and nothing found defective in drainage, ventilation and surroundings, which could give rise to the zymotic condition which supervened. Thorough antiseptic precautions and measures were used. At the time of my attendance upon this patient, erysipelas and diseases of like character were not prevailing in my own or in the practice of my professional brethren. Puerperal fever was not prevailing at that time in the city; the case was a sporadic one. The period of the invasion of the fever is also worthy of note. Seven full days had elapsed and every symptom augured a favorable termination. It was the old teaching upon this subject, only dating back to my pupilage, that five days of convalescence, unattended by symptoms of puerperal fever, assured the patient of an immunity against its invasion. One of the most scholarly of the profession of Buffalo, now deceased—I refer to the late Dr. Winne—was wont to say that the lying-in-woman who passed five days in safety, could rest assured of freedom from puerperal fever; and so deeply impressed has been this precept upon my mind during an experience now quite large, that I have been accustomed to feel a comparative safety when the patient passed over this limit.

So wide a diversity exists in the profession as to the pathology of puerperal fever, the causes leading thereto, the distinctive limitations of the disease, that it may not be surprising, in the case here reported, the writer accepted the situation and remained in fact in a position which was absolutely that of a "looker on," as the case advanced step by step in its attack upon the vital forces, until death claimed its victim.

Passing over the numerous theories, ably upheld by eminent men in the profession, this case impressed one fact upon my mind, that puerperal fever is a disease sui generis, and not necessarily dependent upon any local lesion. What is its specific cause? As well may we attempt to explain the cause of scarlet fever, or any of the other essential fevers, or the specific materies morbi, upon which they depend. Such reflections are naturally suggested, but they lead to a discussion of questions, which it is foreign to my present purpose to enter upon.

The second case here reported presents more complicated and therefore more interesting features. The question of causation and of diagnosis also presents itself. Was it puerperal fever or puerperal septicæmia, or to use the term introduced by Duncan sapræmia? In favor of the diagnosis of puerperal fever, we have the elevated temperature of 102 the evening of the day of her confinement. This was too early for puerperal infection—I mean autogenetic infection. The temperature gradually fell so that on the evening of the fifth day the thermometer registered 100, and the pulse 100. Was this condition due (as it yielded to antipyretic remedies) to zymótic influences? The antecedents of the patient favor this theory. After her fourth pregnancy she had puerperal fever, from which recovery took place after a very severe illness. Her system had been overtaxed by too frequent child-bearing, the present being the seventh child in twelve years, and with each pregnancy there was an increased obesity and therefore diminished resistance or endurance. On the fifth day the patient had the first chill; the lochia had previously assumed a purulent character, though not offensive in odor. The elevated temperatur of 105, which followed, readily yielded to antipyretics and intra-uterine injections of carbolized water, the temperature falling 1 and 2 degrees from the local effect of the antiseptics alone. Here we have symptoms of auto-genetic infection. But let us follow the record further. From the 5th day to the 12th we find a gradual subsidence of the febrile symptoms until on the morning of the 27th—twelve days after delivery—the thermometer registered 99. A few hours after, the second chill occurred. From that time antiseptics, locally, failed to have only a temporary influence; the symptoms becoming more and more grave until her death, which took place on the 19th day. Assuming the position, which I think is well established, that the first chill was due to purulent infection, in a susceptible organization, the subsequent improvement in all the symptoms is explained by the elimination of the poisonous constituents from the blood, the sapræmic or septicæmic phenomena disappearing as the source of supply was cut off. This, indeed, is usually the course. The sapræmia is kept up by a continuous supply of the poison and disappears when the supply is stopped. To stop the supply is the problem of cure. Evidently, in the case under consideration, this problem was not solved, the patient's life succumbing to the disease, notwithstanding the most thorough and energetic use of measures, in the wise administration of which I have the fullest confidence.

I may be permitted to direct attention to a feature of the treatment of this case, which is worthy of note. I refer to intra-uterine antiseptic injections. Much has been written of late upon this subject. Their utility in this case was plainly demonstrated, the temperature always falling under their use, until the system became so thoroughly impregnated with the materies morbi, that the action locally failed to reach the poisoned state of the blood. In their use, I think the attending physician should always exercise caution; 1st, in assuring himself that the os uteri is patulous, and 2d, in avoiding the introduction of air. I think also he should never intrust this procedure to the nurse. It is important that he use them under his own careful guidance and direction.

In recurring to the general subject of septicæmia and sapræmia, it may be well to define the terms. Duncan says that septicæmia is the the result of the growth in the blood of certain micrococci, having the power of rapidly multiplying and thus contaminating the system, while sapræmia is a mere poisoning of the blood by the *chemieal products of putrifaction.* He makes the distinction between the two conditions, the one due to the presence of microscopic germs, and the other due to the *presence* and influence, *chemically* of putrid matter. Other writers fail to make any such distinction, but regard septicæmia to be due to putrid infection. Among these I may include Fordyce Barker, whose work on puerperal diseases is good authority on this continent. But whatever may be the distinctive differences, I think Duncan has drawn a line, which if he is able to substantiate, furnishes a very nice discrimination and an important pathological distinction in these dangerous complications of the puerperal state. He maintains that putridity is not an essential part of septicæmia and pyæmia, though it often occompanies them, and the organisms, which cause these diseases, take no part in putrefaction, living in the discharges and conveyed into the blood where they multiply indefinitely. He also maintains that organisms which cause putrifaction, whether the bacterium termo or others in addition, may pass into the blood to produce sapræmia, but they do not survive, far less grow therein. These are very interesting questions in pathology, which I have not time to discuss. They must be referred to another occasion.

Turning from these general considerations affecting the distinctive differences and pathology of the puerperal conditions above referred to, we must not lose sight of the local *conditions* favoring severe and often fatal complications in the lying-in state; and here, the enlarged absorbing surface, which the uterus offers after delivery, constitutes an important factor. The placental disc, with the often patulous uterine sinuses, the lacerations of the os and cervix, and the abrasions of the vaginal mucous membrane, and lacerations at the vulva, are frequent avenues for the introduction into the system of morbific elements, which acting in a field *especially susceptible from antecedent influences*, work the sad havoc of suffering and death. Nor should we fail to overlook the imperfect contraction of the uterus as a source of danger, and the indications of treatment it presents. These local conditions, as a matter of course, are of no significance unless putrid animal matter is contained in the uterine or vaginal canal. Placental tissue retained, or the debris of the involution of the uterus, which of necessity goes on after parturition, are the most frequent sources of auto-infection, and to these the attention of the accoucheur should be directed. But the uterine and vaginal canal may also be the avenue for the introduction of diseased germs from without. This is termed heterogenetic infection, and is worthy of careful consideration. The digital examination required to determine the stage of labor and presentation of the fœtus, has been the unsuspecting cause of death to many a parturient woman; and if any words of mine can impress upon the members of the Club, the value and the necessity of thorough antisepsis of the hand prior to the performance of this duty, so that hereafter this necessary precaution in all your obstetrical practice shall always be observed, I shall feel that your patients will have an additional safeguard against dangerous complications during the puerperal perod.

An intelligent patient, in labor, once required her attending accoucheur to clean his finger nails before she would permit the digital examination to be made. This patient unconsciously administered a rebuke to her professional attendant, no doubt well deserved, but containing a hint which others may take home to themselves, by extending the cleaning process to the entire hand, and repeating the antiseptic ablutions at each and every digital examination. Let me urge this important measure upon your attention. It is simple, and in the additional safety it brings to the parturient woman, affords ample compensation for the slight trouble it involves. I have sufficiently recommended antiseptic washes to the entire vaginal canal, and, if need be, to the interior of the uterus, to call for no further mention in this connection, and I refer to it again only to emphasize their importance as a prophylactic.

I should also in passing mention the influence of certain seasons, with their atmospheric variations and epidemic conditions, in the causation of puerperal diseases, and also the prevalence of zymotic diseases, as erysipelas and like morbid states, tending to the same end. These are now established factors in the solution of this question, and need not be further discussed in this connection.

The treatment of these morbid conditions would appropriately conclude my paper, if sufficient had not already been written in detailing the history of these cases. It has been well said that cases of septicæmia are to be managed rather than treated. If the attack is due to the presence of putrid matter in the uterus, the physician has it in his power to strike a blow which, if done promptly before the system has become contaminated, will effectually arrest the disease. The use of antipyretics, as quiniæ sulph., salicylic acid, aconite, etc., are indicated; the strictest attention to cleanliness, ventilation, to the diet, that it may afford support to the system rapidly exhausting its forces under the influence of combustion going on within, to prudent stimulation, etc. We may go further and refer to tincture of iron and chlorate of potash in septicæmia or sapræmia; but these general directions must guide the physician who unfortunately may have to encounter what has proven to me the most unpleasant, the saddest experience of my professional life.

